# Exogenous Ketone Supplement Administration Abrogated Isoflurane-Anesthesia-Induced Increase in Blood Glucose Level in Female WAG/Rij Rats

**DOI:** 10.3390/nu16101477

**Published:** 2024-05-14

**Authors:** Enikő Rauch, Csilla Ari, Dominic P. D’Agostino, Zsolt Kovács

**Affiliations:** 1 Department of Biology, Berzsenyi Dániel Teacher Training Centre, ELTE Eötvös Loránd University, Károlyi Gáspár tér 4, 9700 Szombathely, Hungary; raucheniko9810@gmail.com (E.R.);; 2 Institute of Biology, University of Pécs, Ifjúság Str. 6, 7624 Pécs, Hungary; 3 Ketone Technologies LLC, Tampa, FL 33612, USA; ddagosti@usf.edu; 4 Behavioral Neuroscience Research Laboratory, Department of Psychology, University of South Florida, Tampa, FL 33620, USA; 5 Laboratory of Metabolic Medicine, Department of Molecular Pharmacology and Physiology, Morsani College of Medicine, University of South Florida, Tampa, FL 33612, USA; 6 Institute for Human and Machine Cognition, Ocala, FL 34471, USA

**Keywords:** isoflurane anesthesia, ketone supplement, glucose level, recovery time, adenosine receptor, female WAG/Rij rat

## Abstract

It has been demonstrated that isoflurane-induced anesthesia can increase the blood glucose level, leading to hyperglycemia and several adverse effects. The administration of a mix of ketone diester (KE) and medium-chain triglyceride (MCT) oil, named KEMCT, abolished the isoflurane-anesthesia-induced increase in blood glucose level and prolonged the recovery time from isoflurane anesthesia in a male preclinical rodent model, Wistar Albino Glaxo/Rijswijk (WAG/Rij) rats. While most preclinical studies use exclusively male animals, our previous study on blood glucose changes in response to KEMCT administration showed that the results can be sex-dependent. Thus, in this study, we investigated female WAG/Rij rats, whether KEMCT gavage (3 g/kg/day for 7 days) can change the isoflurane (3%)-anesthesia-induced increase in blood glucose level and the recovery time from isoflurane-evoked anesthesia using the righting reflex. Moreover, KEMCT-induced ketosis may enhance both the extracellular level of adenosine and the activity of adenosine A1 receptors (A1Rs). To obtain information on the putative A1R mechanism of action, the effects of an A1R antagonist, DPCPX (1,3-dipropyl-8-cyclopentylxanthine; intraperitoneal/i.p. 0.2 mg/kg), on KEMCT-generated influences were also investigated. Our results show that KEMCT supplementation abolished the isoflurane-anesthesia-induced increase in blood glucose level, and this was abrogated by the co-administration of DPCPX. Nevertheless, KEMCT gavage did not change the recovery time from isoflurane-induced anesthesia. We can conclude that intragastric gavage of exogenous ketone supplements (EKSs), such as KEMCT, can abolish the isoflurane-anesthesia-induced increase in blood glucose level in both sexes likely through A1Rs in WAG/Rij rats, while recovery time was not affected in females, unlike in males. These results suggest that the administration of EKSs as an adjuvant therapy may be effective in mitigating metabolic side effects of isoflurane, such as hyperglycemia, in both sexes.

## 1. Introduction

Inhalational anesthetic isoflurane (1-chloro-2,2,2-trifluoroethyl difluoromethyl ether) crosses the blood–brain barrier (BBB) and neuronal membranes, leading to not only general anesthesia, but also neuroprotective influences [1,2,3]. For example, it has been demonstrated that isoflurane can inhibit apoptosis, reduce excitotoxicity, prevent mitochondrial dysfunction and have anti-inflammatory influence [4,5,6]. Consequently, isoflurane may generate alleviating effects in the treatment of several central nervous system diseases, such as cerebral ischemia, epilepsy, depression and Alzheimer’s disease [3,7,8]. Moreover, isoflurane can modulate metabolic processes in the brain, including cortical metabolism [9]. It has also been demonstrated that the administration of isoflurane [2,10] can increase the blood glucose level through enhanced glucose production and reduced glucose clearance [11,12], leading to hyperglycemia as a consequence [13]. Moreover, hyperglycemic effects can decrease the cardioprotective effect of isoflurane [14,15]. Consequently, theoretically, a decrease in isoflurane-induced hyperglycemic effects may allow us to not only avoid adverse side effects but also preserve the potential protective effects of isoflurane.

It was also previously demonstrated that different exogenous ketone supplements (EKSs) and their combinations, such as the mix of ketone diester (KE) and medium-chain triglyceride (MCT) oil (named KEMCT), may enhance and maintain the blood ketone body level (e.g., R-β-hydroxybutyrate, R-βHB) and reduce the level of blood glucose not only in animals but also in humans [16,17,18]. These effects of EKSs were generated not only under physiological but also in pathological conditions, such as epilepsy and anxiety [16,19,20]. Furthermore, it was suggested that EKSs can modulate both sleep-like influences and isoflurane anesthesia [21,22]. Consequently, it was hypothesized that EKSs would reduce isoflurane-anesthesia-evoked changes in blood glucose level and recovery time. Indeed, the administration of KEMCT by intragastric gavage enhanced the blood R-βHB level and reduced the blood glucose level, as well as decreased the isoflurane-anesthesia-evoked increase in blood glucose level and prolonged the time required for recovery from isoflurane-generated anesthesia in an animal model of human absence epilepsy, Wistar Albino Glaxo Rijswijk (WAG/Rij) rats [23]. Moreover, KEMCT treatment delayed the onset of isoflurane-generated anesthesia in WAG/Rij rats [24]. These results suggest that, theoretically, the administration of EKSs as an adjuvant therapy may not only alter isoflurane-induced anesthesia but also attenuate isoflurane-anesthesia-generated side effects, such as hyperglycemia, not only in animal models but also in humans.

It has also been demonstrated that an EKS-generated increase in ketone body levels can enhance the level of adenosine in the brain [25,26], leading to enhanced activity of adenosine A1 receptors (A1Rs) and neuronal hyperpolarization [27,28]. These last effects may modulate the influences evoked by EKSs, such as KEMCT, on the isoflurane-generated onset of anesthesia and, theoretically, on the isoflurane-evoked increase in both glucose level and recovery time from isoflurane anesthesia [23,24,29]. Although the KEMCT-induced reduction in blood glucose level and increase in R-βHB level is sex-dependent in WAG/Rij rats [30], the influence of KEMCT administration on isoflurane-anesthesia-induced changes in blood R-βHB and glucose levels and recovery time were investigated only in males so far, but not in female WAG/Rij rats [23].

While most preclinical studies use exclusively male animals, our previous study on blood glucose changes in response to KEMCT administration shows that the results can be sex-dependent. It appears that KEMCT affects female and male WAG/Rij rats differently and that these differences are also influenced by age [30]. In that earlier study, KEMCT gavage induced significantly lower glucose levels at the 4th, 7th, 9th, 10th, 12th, and 13th months in females, whereas it induced significantly lower glucose levels between the 4th and 6th months and between the 9th and 13th months in males, compared with the results at the 1st month. In addition, KEMCT treatment induced lower blood glucose levels in female rats than in male rats between the 1st and 8th months, but significantly higher glucose levels were measured in female rats at the 17th month than in males. These results suggest that blood glucose level changes may be significantly different between female and male rats in response to KEMCT treatment during isoflurane anesthesia. Thus, in this study, we extended our previous experiments on ten-month-old males [23] to similar-age female WAG/Rij rats.

Thus, as a continuation of our previous study on male WAG/Rij rats [23], we investigated whether (i) KEMCT administration can generate changes in isoflurane-evoked alterations in blood glucose level and recovery time from anesthesia in female WAG/Rij rats (putative sex-dependent difference) and (ii) A1Rs can modulate EKS- and isoflurane-anesthesia-evoked effects in female WAG/Rij rats. In accordance with the main goals of this study, we investigated (i) the KEMCT administration (gavage, 3 g/kg once a day for 7 days)-generated effects on isoflurane (3%)-anesthesia-evoked influences on blood glucose and R-βHB levels, as well as recovery time from anesthesia, and (ii) the influence of an A1R antagonist, DPCPX (1,3-dipropyl-8-cyclopentylxanthine; intraperitoneal/i.p. administration of 0.2 mg/kg), on the above-mentioned KEMCT-evoked putative effects by assessing the righting reflex in female WAG/Rij rats. We hypothesized that KEMCT administration can abolish the isoflurane-induced increase in blood glucose level and alter recovery time from anesthesia through A1Rs in female rats.

## 2. Methods

### 2.1. Experimental Animals

Experiments on animals were performed according to (i) the Hungarian Act of Animal Care and Experimentation (1998, XXVIII, section 243), European Communities Council Directive (86/609; EEC) and EU Directive (2010/63; EU), as well as (ii) approval of the Animal Care and Experimentation Committee (Savaria University Centre, Eötvös Loránd University) and National Scientific Ethical Committee on Animal Experimentation (license: VA/ÉBÁF-ÁO/00279-4/2021; Hungary).

Ten-month-old female WAG/Rij rats (*n* = 56; 182–212 g) were housed in groups containing 4 animals in each group (breeding colony in Savaria University Centre, Eötvös Loránd University) under standard laboratory conditions (light–dark cycle 12:12 h; with light on between 08.00 A.M. and 08.00 P.M.; ad libitum access to food/SSNIFF RM-Z+H rat breeding and maintenance diet, by TOXI-COOP Ltd., Budapest, Hungary, and water; room temperature kept at 22 ± 2 °C). On the last day of experiments, the rats were humanely euthanized using isoflurane. All efforts were made to minimize pain, suffering and the number of animals used in the study.

### 2.2. Experimental Design

It has been demonstrated in our previous studies that gavage of 3 g/kg KEMCT for 7 days not only increased the blood R-βHB level, but also reduced the blood glucose level effectively without side effects, such as diarrhea [23]. Thus, similarly to our previous studies on WAG/Rij rats [23,24], in the present study, we fed all rats with a standard rodent chow diet, supplemented with KEMCT (3 g/kg/day) through intragastric needle (gavage) for 7 days. KEMCT contained KE (1,3-butanediol-acetoacetate diester), which was mixed with MCT oil (≈60% caprylic triglyceride and 40% capric triglyceride) in a 1:1 ratio (purchased from University of South Florida, Tampa, FL, USA; Savind, Inc. Urbana, IL, USA; Now Foods, Bloomingdale, IL, USA).

**Figure 1:**
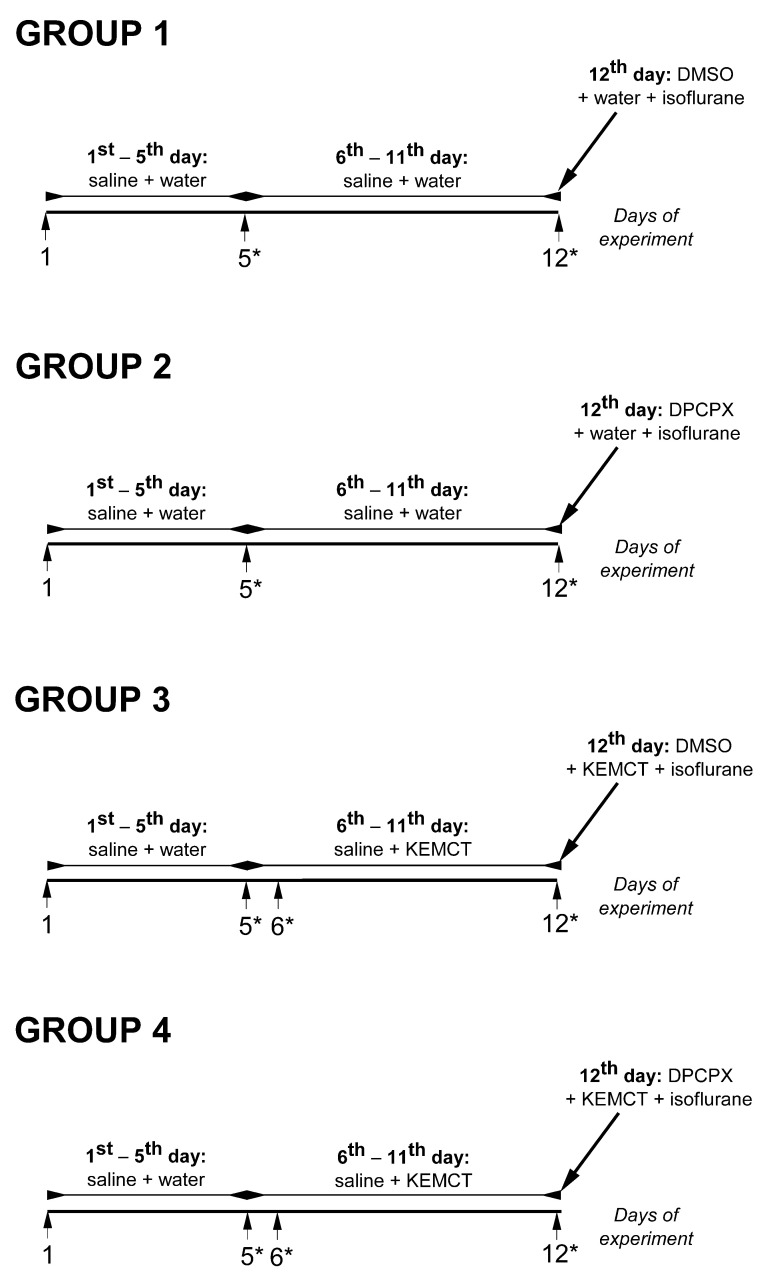
Details of the experimental design. Abbreviations: *, days of blood R-βHB and glucose level measurement; DMSO, dimethyl sulfoxide; DPCPX, 1,3-dipropyl-8-cyclopentylxanthine; KEMCT, mix of KE (1,3-butanediol-acetoacetate diester) and MCT oil (≈60% caprylic triglyceride + 40% capric triglyceride) in 1:1 ratio.

To adapt the animals for treatment methods, i.p. injection of saline (1 mL/kg) and, half an hour later, water gavage (3 g/kg) were administered for 5 days (adaptation period) to female WAG/Rij rats (Figure 1). After the adaptation period, rats were assigned into 4 groups (14 animals/group). Animals of the first and second groups (group 1 and group 2) were i.p. injected with saline (1 mL/kg) and, after 30 min, treated with water gavage between the 6th and 11th day of experiments. On these same days, rats of the third and fourth groups (group 3 and group 4) were treated with KEMCT gavage 30 min after the i.p. saline (1 mL/kg) (Figure 1). On the 12th day of experiments, animals of group 1 received 1 mL/kg 10% dimethyl sulfoxide (DMSO) solution i.p. and, after 30 min, water gavage. Animals of group 2 received similar treatments to animals in group 1, but the DMSO solution contained 0.2 mg/kg DPCPX (group 2). Animals in group 3 were treated with i.p. 1 mL/kg 10% DMSO solution, and 30 min later, KEMCT gavage was administered, whereas animals in group 4 received KEMCT 30 min after the i.p. injection of 0.2 mg/kg DPCPX in 10% DMSO solution.

In relation to all animal groups (group 1–group 4), 60 min after the last gavage, an isoflurane (3%)–air mixture was applied to evoke general anesthesia in an airtight anesthesia chamber for 20 min, as previously described [23]. The righting reflex was used to measure the time required for recovery from isoflurane-induced anesthesia immediately after 20 min of anesthesia. The righting reflex is a well-known postural response of animals by which animals are able to reorient themselves when placed on their side or back. As a result, after recovery of the righting reflex from (isoflurane) anesthesia, the animals’ paws will be reoriented to the ground again, which has been considered as an indicator of emergence from anesthesia in previous studies [1,31,32]. In this study, after 20 min anesthesia, the rats were placed on their backs in a Plexiglas box, and the recovery time (or emergence time/period: the time between termination of isoflurane anesthesia and recovery of the righting reflex) was video recorded and determined by a blinded observer [23].

### 2.3. Blood R-βHB and Glucose Levels, as Well as Body Weight

The blood levels of glucose and R-βHB were measured by a ketone and glucose monitoring system (Precision Xtra™, Abbott Laboratories, Green Oaks, IL, USA) from the blood taken from the tail vein of rats [23]. The measuring of blood R-βHB and glucose levels was carried out 90 min after the gavage on the 5th (control) and 12th (last) days (group 1 and group 2) or the 5th (control), 6th and 12th (last) days (group 3 and group 4) of experiments (Figure 1). On day 12, the blood levels of R-βHB and glucose were measured several minutes after recovery from isoflurane-induced anesthesia.

Body weight was measured on the 5th day (control) and on the 12th day of experiments (group 3 and group 4).

### 2.4. Statistics

The mean and the standard error of the mean (S.E.M.) were used for data presentation. The levels of blood glucose and R-βHB and body weight were compared to control values (measured on the 5th day of experiments). In relation to recovery time from isoflurane anesthesia, results from group 1 (1 mL/kg 10% DMSO solution i.p. + 3 g/kg water gavage) were compared to group 3 (1 mL/kg 10% DMSO solution i.p. + 3 g/kg KEMCT gavage), whereas results from group 2 (0.2 mg/kg DPCPX in 1 mL/kg 10% DMSO solution i.p. + 3 g/kg water gavage) were compared with group 4 (0.2 mg/kg DPCPX in 1 mL/kg 10% DMSO solution i.p. + 3 g/kg KEMCT gavage). Two-way ANOVA, Tukey’s multiple comparisons test, Šídák’s multiple comparisons test and *t*-test were performed using GraphPad version 9.2.0. [23]. Statistical significance was accepted when *p* < 0.05.

## 3. Results

### 3.1. Influence of DPCPX and KEMCT on Isoflurane-Anesthesia-Evoked Alterations in Level of Blood Glucose and R-βHB and on Body Weight

**Figure 2:**
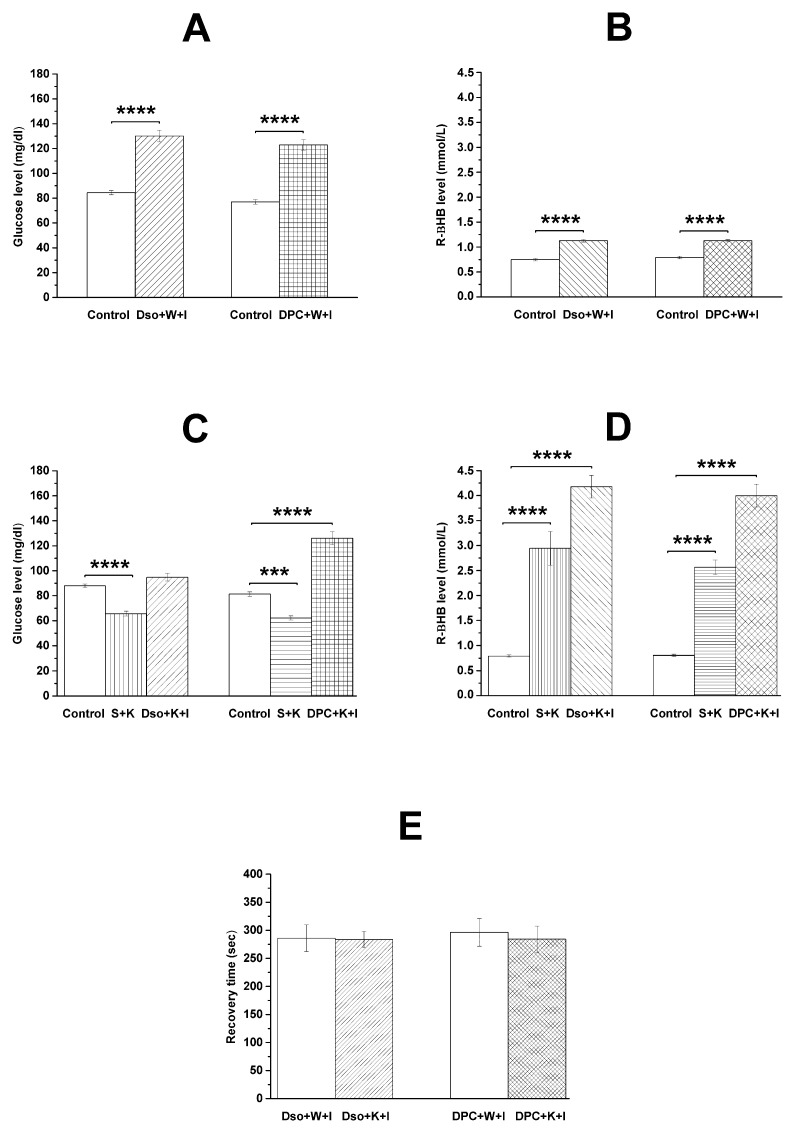
KEMCT-, DPCPX- and their combination-generated effects on isoflurane-anesthesia-evoked changes in blood glucose (**A**,**C**) and R-βHB (**B**,**D**) levels, as well as recovery time (**E**), compared to control. Abbreviations: DPC+K+I, group 4 (0.2 mg/kg DPCPX in 1 mL/kg 10% DMSO solution i.p. + 3 g/kg KEMCT gavage + isoflurane anesthesia); DPC + W + I, group 2 (0.2 mg/kg DPCPX in 1 mL/kg 10% DMSO solution i.p. + 3 g/kg water gavage + isoflurane anesthesia); Dso + K + I, group 3 (1 mL/kg 10% DMSO solution i.p. + 3 g/kg KEMCT gavage + isoflurane anesthesia); Dso + W + I, group 1 (1 mL/kg 10% DMSO solution i.p. + 3 g/kg water gavage + isoflurane anesthesia); R-βHB, R-beta-hydroxybutyrate; S + K, group 3 and group 4 (1 mL/kg saline i.p. + 3 g/kg KEMCT gavage); *** *p* < 0.001; **** *p* < 0.0001.

Isoflurane-generated anesthesia significantly enhanced the blood level of both glucose and R-βHB compared to the control (left part of Figure 2A,B; Table 1: group 1). We also demonstrated that the administration of i.p. 0.2 mg/kg DPCPX alone (without KEMCT) was not able to change the isoflurane-generated effects on blood glucose and R-βHB levels (right parts of Figure 2A,B; Table 1: group 2).

On the 6th experimental day, the first KEMCT gavage significantly decreased the blood level of glucose and increased the level of R-βHB compared to the control (Figure 2C,D; Table 1: group 3 and group 4). Nevertheless, the seventh gavage of KEMCT (on the 12th day of experiments) alone (without DPCPX) was able not only to increase the blood R-βHB level more efficiently (left part of Figure 2D; Table 1: group 3), but also to maintain the normal (control) level of blood glucose under isoflurane-generated anesthesia (left part of Figure 2C). Namely, the isoflurane-anesthesia-induced increase in blood glucose level was abolished by administration of KEMCT compared to the control in female WAG/Rij rats (left part of Figure 2C; Table 1: group 3), which is similar to our previous results for male WAG/Rij rats [23]. Moreover, i.p. 0.2 mg/kg DPCPX abolished the seventh KEMCT-gavage-evoked alleviating effect on the isoflurane-induced increase in blood glucose level. Thus, under these circumstances, the level of blood glucose significantly increased compared to the control (right part of Figure 2C; Table 1: group 4). However, DPCPX did not alter the KEMCT-generated increase in blood R-βHB level (Figure 2D; Table 1: group 4).

Similar to our previous study on male WAG/Rij rats [23], the body weight of female WAG/Rij rats was not changed compared to the control (control/KEMCT alone, group 3: 200.5 ± 1.82 g/199.1 ± 2.50 g, *p* = 0.5778; control/DPCPX + KEMCT, group 4: 191.6 ± 1.57 g/190.1 ± 2.01 g, *p* = 0.5472).

### 3.2. Effects of DPCPX and KEMCT on Recovery Time

In contrast to our previous study on male WAG/Rij rats [23], recovery time from isoflurane-evoked anesthesia did not change after KEMCT gavage alone or combined administration of KEMCT gavage with DPCPX (0.2 mg/kg i.p.) in female WAG/Rij rats (DMSO + water + isoflurane/group 1 v. DMSO + KEMCT + isoflurane/group 3: *p* = 0.9312; DPCPX + water + isoflurane/group 2 v. DPCPX + KEMCT + isoflurane/group 4: *p* = 0.7225) (Figure 2E).

## 4. Discussion

As putative sex-dependent effects of EKSs can have important implications during anesthesia, we studied and compared females to our previous study on male rats. In this study, we investigated the KEMCT-administration-generated effects on isoflurane-induced changes in blood glucose level and recovery time using female WAG/Rij rats. We demonstrated that like in male WAG/Rij rats [23], isoflurane anesthesia (i) can increase blood glucose and R-βHB levels; KEMCT administration (ii) was able to abolish the isoflurane-anesthesia-induced augmentation in blood glucose level, whereas (iii) it did not alter the body weight of female rats. These results strengthened our previous results on male WAG/Rij rats and suggested sex-independent effects of EKSs. Moreover, we also demonstrated a sex-dependent effect of KEMCT administration: in contrast to our previous results on male WAG/Rij rats, KEMCT gavage did not alter recovery time from isoflurane-generated anesthesia. We extended our previous results, as we also suggested that (i) KEMCT administration may evoke its beneficial effect on the isoflurane-anesthesia-induced increase in blood glucose level likely through a ketosis-induced increase in adenosine level and A1R activity in the brain and (ii) inhibition of A1Rs alone did not change the isoflurane-anesthesia-induced effects on blood glucose and R-βHB levels.

Earlier studies suggest that insulin resistance and hyperglycemia may be related to, among others, immunosuppression, cardiovascular problems and ischemic brain damage [13,33,34]. Isoflurane anesthesia may impair glucose clearance and produce hyperglycemia likely through processes evoked by ATP-sensitive potassium channels in pancreatic β-cells leading to a decrease in insulin release and glucose utilization [35,36]. Indeed, it was evidenced that isoflurane anesthesia increased the blood glucose level in both male [23] and female WAG/Rij rats (left part of Figure 2A), and this influence was abolished by KEMCT administration [23] (left part of Figure 2C). In relation to the putative mechanism of action of the KEMCT-evoked influence on isoflurane-anesthesia-generated effects on blood glucose levels, it was demonstrated that EKSs are able to increase the blood ketone body level as well as mitigate the blood glucose level [23,37], suggesting that EKS-evoked ketosis is able to increase insulin sensitivity [38]. It has also been demonstrated that insulin sensitivity and glucose tolerance were impaired in A1R KO mice [39], whereas A1R overexpression can protect mice from insulin resistance [40]. Furthermore, A1R activation can increase insulin sensitivity [41,42]. Thus, as KEMCT-induced ketosis can increase the adenosine level in the brain [25,26], it is possible that KEMCT could modulate the effect of isoflurane anesthesia on the blood glucose level through A1Rs. Indeed, we demonstrated that DPCPX alone was not effective on the isoflurane-anesthesia-induced elevation in blood glucose level (right part of Figure 2A), but i.p. injection of DPCPX in combination with KEMCT gavage abrogated the beneficial influence of KEMCT treatment on isoflurane-anesthesia-generated changes in blood glucose levels (Figure 2C).

We demonstrated previously that isoflurane anesthesia alone, KEMCT alone and the combination of KEMCT gavage with isoflurane anesthesia increased the blood R-βHB level in male WAG/Rij rats [23]. These results were strengthened by the present study in female WAG/Rij rats (left parts of Figure 2B,D), suggesting that the isoflurane anesthesia increases the blood R-βHB level sex-independently. Moreover, KEMCT administration can enhance the R-βHB level of blood under isoflurane-induced anesthesia, also independently of sex. These effects on the blood R-βHB level evoked by the administration of isoflurane alone and isoflurane in combination with KEMCT were not modulated by DPCPX (right parts of Figure 2B,D), revealing that A1Rs likely do not have a role in these processes, at least in female WAG/Rij rats.

Previous studies suggest that adenosine and A1Rs may modulate the recovery time from isoflurane-evoked anesthesia. For example, not only non-selective antagonists of adenosine receptors, such as caffeine and theophylline in both rats and mice [43,44,45], but also DPCPX in mice decreased the time spent to recover from isoflurane anesthesia [44]. Nevertheless, an A1R agonist, N-p-sulfophenyl adenosine, augmented the recovery time from isoflurane-evoked anesthesia in mice, likely through A1Rs [29]. Although KEMCT-administration-evoked ketosis can exert its effects on isoflurane-anesthesia-evoked influences through increased adenosine levels and A1R activity [25,26,43,44,45] and KEMCT administration for 7 days prolonged the recovery time from isoflurane anesthesia in male WAG/Rij rats, perhaps through A1Rs [23], in this study, the administration of KEMCT alone (group 3) did not alter the recovery time in female WAG/Rij rats (left part of Figure 2E). It was demonstrated previously that metabolic enzymes and processes modulating the adenosine level and, consequently, the extracellular level of adenosine, as well as the expression of nucleoside transporters and activity of A1Rs in the brain, are sex-dependent [46,47,48]. Thus, these results suggest that the effect of KEMCT administration on the time required to recover from isoflurane anesthesia may be sex-dependent, likely through the adenosinergic system, at least in WAG/Rij rats. Nonetheless, it was demonstrated that the recovery time from isoflurane anesthesia was similar in male and female rats [49] and was not modulated by the estrus cycle [50].

We demonstrated previously that the administration of different i.p. doses of DPCPX (0.15–0.25 mg/kg) alone was not effective on absence epileptic activity, anxiety level and the onset of the isoflurane-evoked light phase of anesthesia [19,20,24]. Nevertheless, the administration of the same doses of DPCPX in combination with EKSs, such as KEMCT, KSMCT (mix of ketone salt/KS and MCT oil) and/or KEKS (mix of KE and KS), was effective in the inhibition of EKS-generated effects on absence epileptic activity (0.2 mg/kg DPCPX i.p.), anxiety (0.15 and 0.25 mg/kg DPCPX i.p.) and the onset of the isoflurane-generated light phase of anesthesia (0.2 mg/kg DPCPX i.p.) in male WAG/Rij rats [19,20,24]. Similarly, in this study, DPCPX alone (group 2) was ineffective in changing the recovery time from isoflurane anesthesia in female WAG/Rij rats (right part of Figure 2E). Nevertheless, based on (i) the above-mentioned results suggesting that the applied dose of DPCPX could be effective only in combination with EKSs if the administration of KEMCT alone was effective, for example, in the modulation of isoflurane-evoked effects [19,20,24] and (ii) the lack of efficacy of KEMCT gavage alone (group 3) on recovery time (left part of Figure 2E), it is not astonishing that combined administration of DPCPX and KEMCT (group 4) was also not effective in changing the recovery time (right part of Figure 2E). However, higher doses of KEMCT may change the time required to recover from isoflurane anesthesia. Moreover, there may be a need to test whether different doses and types of EKSs evoke significant effects on different physiological and pathophysiological processes under different circumstances in male and female rats [30] through the interaction of several neurotransmitter systems (e.g., adenosinergic, serotonergic and GABAergic system) and receptors [51]. Thus, further studies are needed to investigate the effect of different doses and types of EKSs, as well as their exact mechanism of action, on not only the isoflurane-evoked increase in blood glucose level but also the recovery time from isoflurane-induced anesthesia through different neurotransmitter systems, such as the adenosinergic system and A1Rs.

## 5. Conclusions

Based on our earlier study on male WAG/Rij rats, together with the present study performed on female WAG/Rij rats, we can conclude that isoflurane-induced anesthesia can increase the blood level of both glucose and R-βHB independently of sex. Moreover, KEMCT administration was able to abolish the isoflurane-anesthesia-evoked increase in blood glucose level, also independently of sex, likely through A1Rs. Nevertheless, unlike the results obtained on male WAG/Rij rats, KEMCT gavage did not change the recovery time from isoflurane-evoked anesthesia in female WAG/Rij rats, suggesting a sex-dependent influence. These results further strengthened our previous suggestion that the administration of EKSs (e.g., KEMCT) as an adjuvant therapy may be effective in mitigating the metabolic side effects of isoflurane, such as hyperglycemia, in both sexes. However, further studies are needed to reveal the putative sex-dependent effects, as well as the exact mechanism of action, of EKSs on influences evoked by isoflurane and other types of anesthesia in both males and females.

## Figures and Tables

**Table 1:** Effects of KEMCT gavage and i.p. DPCPX on isoflurane-anesthesia-generated changes in blood R-βHB and glucose levels, compared to control. Abbreviations: ns, non-significant; R-βHB, R-beta-hydroxybutyrate; *** *p* < 0.001; **** *p* < 0.0001.

Treatments	Glucose (mg/dL)	R-βHB (mmol/L)
Group 1		
Control	84.43 ± 1.775	0.75 ± 0.027
12th day of experiments	130.07 ± 4.748 ****/<0.0001	1.13 ± 0.027 ****/<0.0001
Group 2		
Control	77.00 ± 1.816	0.79 ± 0.027
12th day of experiments	123.00 ± 4.235 ****/<0.0001	1.13 ± 0.022 ****/<0.0001
Group 3		
Control	88.00 ± 1.371	0.79 ± 0.019
6th day of experiments	65.71 ± 1.962 ****/<0.0001	2.94 ± 0.338 ****/<0.0001
12th day of experiments	94.79 ± 3.015 ns/0.0913	4.18 ± 0.228 ****/<0.0001
Group 4		
Control	81.36 ± 1.833	0.80 ± 0.019
6th day of experiments	62.29 ± 1.588 ***/0.0005	2.56 ± 0.146 ****/<0.0001
12th day of experiments	126.14 ± 5.122 ****/<0.0001	4.00 ± 0.228 ****/<0.0001

## Data Availability

The data presented in this study are available on request from the corresponding author. The data are not publicly available due to ethical reasons.

## Abbreviations

A1R: adenosine A1 receptor; DMSO, dimethyl sulfoxide; DPCPX, 1,3-dipropyl-8-cyclopentylxanthine; EKS, exogenous ketone supplement; i.p., intraperitoneal; KE (ketone diester), 1,3 butanediol-acetoacetate diester; KEMCT, mix of KE and MCT oil; MCT, medium-chain triglyceride; R-βHB, R-beta-hydroxybutyrate; WAG/Rij, Wistar Albino Glaxo/Rijswijk.
